# Diagnostic Accuracy of Post-Treatment ADC and MRI Response Assessment for Predicting Pathological Complete Response in Breast Cancer Patients Receiving Neoadjuvant Chemotherapy

**DOI:** 10.3390/jcm15135026

**Published:** 2026-06-27

**Authors:** Ela Kaplan, Hüseyin Alakus, Selcuk Kaplan

**Affiliations:** 1 Department of Radiology, Faculty of Medicine, Adiyaman University, Adıyaman 02040, Turkey; 2 Departmant of Surgery Oncology, Faculty of Medicine, Adiyaman University, Adıyaman 02040, Turkey; dr.alakus@hotmail.com; 3 Departmant of Gynecology and Obstetrics, Faculty of Medicine, Adiyaman University, Adıyaman 02040, Turkey; kaplan_2384@hotmail.com

**Keywords:** breast neoplasms, diffusion magnetic resonance imaging, neoadjuvant therapy, treatment outcome

## Abstract

**Background/Objectives**: This study aimed to evaluate the diagnostic performance of post-neoadjuvant chemotherapy (NAC) contrast-enhanced MRI in breast cancer treatment response assessment and to determine whether apparent diffusion coefficient (ADC) parameters contribute to predicting and confirming pathologic complete response (pCR). **Methods:** This retrospective cohort study enrolled patients with histopathologically confirmed invasive breast cancer who underwent breast MRI before and after NAC, followed by surgical resection. Post-NAC MRI response was classified into four categories and subsequently dichotomised into complete response versus residual disease. On diffusion-weighted imaging (DWI), pre-treatment minimum and maximum ADC values, post-treatment ADC, and the percentage change in ADC (ΔADC) were calculated. Diagnostic performance was assessed by sensitivity, specificity, positive predictive value (PPV), negative predictive value (NPV), receiver operating characteristic (ROC) curve analysis, and Cohen’s kappa coefficient. **Results:** Of 188 patients, 19.7% achieved pCR. Post-NAC MRI complete response predicted pCR with 100% sensitivity and 90.1% specificity, with a Cohen’s kappa of 0.781 and 15 false-positive cases. Post-treatment ADC achieved the highest predictive performance, with an area under the ROC curve (AUC) of 0.967 (optimism-corrected 0.958); at the derived threshold, sensitivity was 100.0% and specificity 96.7%. ΔADC was likewise a statistically significant predictor of pCR, and post-treatment ADC remained independent after adjustment. Pre-treatment ADC parameters carried no meaningful predictive value in the full-cohort analysis. **Conclusions:** Post-treatment ADC and ΔADC help identify true complete responders among cases that contrast-enhanced MRI alone misclassifies as positive. Incorporating quantitative diffusion parameters into standard post-NAC MRI assessment may support pCR confirmation and warrants prospective external validation.

## 1. Introduction

Breast cancer remains the most common malignancy in women worldwide, with 2.3 million new cases recorded in 2022 according to GLOBOCAN data [1]. Neoadjuvant chemotherapy (NAC) is the standard approach in locally advanced disease, achieving pathologic complete response (pCR) in 6–25% of patients; those who attain pCR carry a meaningfully better overall survival [2]. Contrast-enhanced MRI (CE-MRI) has become the dominant non-invasive modality for monitoring treatment response—though the evidence base for this position is less settled than its routine use might suggest. A meta-analysis spanning 54 studies found specificity reaching 0.92, yet sensitivity stalled at 0.64 [3]. Diffusion-weighted imaging (DWI) outperformed CE-MRI with an area under the curve (AUC) of 0.94, and a series of 1062 patients identified 1.22 × 10^−3^ mm^2^/s as the optimal post-NAC apparent diffusion coefficient (ADC) threshold for distinguishing residual disease from complete response [4].

The limited sensitivity of CE-MRI traces back to several converging problems: fibrosis, non-mass enhancement (NME), and peritumoural oedema all generate false-positive signals, while size measurement inconsistencies vary by molecular subtype [3,4]. An individual patient data meta-analysis found that MRI–pathology agreement limits extended to ±3.8 cm—a discrepancy that directly calls into question the reliability of CE-MRI as a standalone decision-making tool [5]. Meta-regression of dynamic contrast-enhanced MRI studies further revealed that no covariate could account for the I^2^ = 58–65% heterogeneity across studies, pointing to a lack of acquisition and interpretation standardisation as the root of threshold instability [2,6]. Pre-treatment ADC has shown considerable overlap between responders and non-responders. The percentage change in ADC (ΔADC%) appears more discriminating, but sample sizes ranging from 24 to 174 patients and the absence of subtype-specific cut-off values have limited its clinical translation [1]. Models combining MRI with Ki-67 carry the same problem in a different form—I^2^ = 77% and unresolved protocol heterogeneity [7].

Against this backdrop, studies that evaluate post-NAC CE-MRI accuracy together with post-treatment ADC and ΔADC, while accounting for molecular subtype differences, remain underrepresented in the literature. The biological rationale is straightforward: tumour cell death in cases achieving pCR increases free water diffusivity, which should raise both post-treatment ADC and ΔADC and, in principle, help resolve the false-positive cases that CE-MRI cannot. The stakes are highest in exactly these patients—the ones CE-MRI judges to be complete responders—because the error that matters here is not over-treatment but its opposite: when CE-MRI reports a clear tumour bed while residual disease is in fact present, the real hazard is that the patient is undertreated or wrongly considered for de-escalation, not that surgery is carried out needlessly. Any step in that direction would first have to be confirmed in prospective, externally validated cohorts. For that reason we approached post-treatment ADC primarily as a confirmatory step in this group rather than as a general post-NAC biomarker. We therefore set out to assess the diagnostic accuracy of post-NAC CE-MRI for confirming pCR and to determine whether ADC and ΔADC contribute independently to distinguishing pCR from residual disease and whether applying a post-treatment ADC threshold to the CE-MRI complete responders could correctly reclassify the false positives without missing any true responders. We evaluated these questions using sensitivity, specificity, positive predictive value (PPV), negative predictive value (NPV), and receiver operating characteristic (ROC) parameters as the primary benchmarks.

## 2. Materials and Methods

### 2.1. Study Population

**Figure 1:**
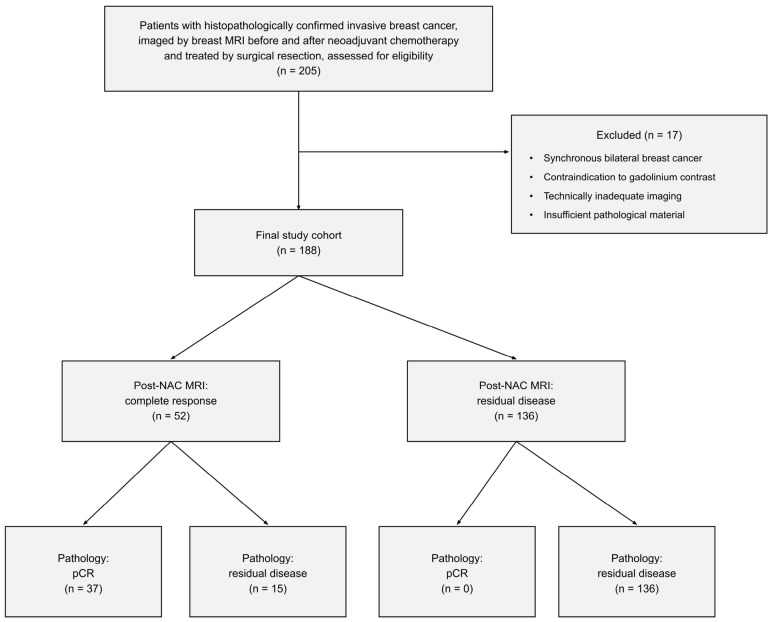
Study flow diagram showing participant selection, exclusion, allocation by post-treatment MRI response, and cross-classification against surgical pathology. Of 205 patients with histopathologically confirmed invasive breast cancer imaged by breast MRI before and after neoadjuvant chemotherapy and treated by surgical resection, 17 were excluded (synchronous bilateral breast cancer, contraindication to gadolinium contrast, technically inadequate imaging, or insufficient pathological material), leaving a final cohort of 188. Patients were grouped by post-treatment MRI response (complete response, *n* = 52; residual disease, *n* = 136) and cross-classified against surgical pathology (complete response: pCR, *n* = 37, and residual disease, *n* = 15; residual disease: pCR, *n* = 0, and residual disease, *n* = 136). MRI, magnetic resonance imaging; NAC, neoadjuvant chemotherapy; pCR, pathological complete response.

This single-centre retrospective cohort study enrolled patients with histopathologically confirmed invasive breast cancer who had undergone breast MRI both before and after neoadjuvant chemotherapy (NAC) and subsequently proceeded to surgical resection. Consecutive patients managed at our institution between January 2017 and December 2023 were screened for eligibility. Patients with synchronous bilateral breast cancer, a contraindication to gadolinium-based contrast agents, technically inadequate imaging, or insufficient pathological material were excluded. Of 205 patients assessed for eligibility, 17 met an exclusion criterion, leaving 188 patients in the final analysis (Figure 1). Age, maximum tumour diameter, Ki-67 proliferation index, and receptor status—oestrogen receptor (ER), progesterone receptor (PR), and human epidermal growth factor receptor 2 (HER2)—were retrieved from medical records. Post-treatment ADC was evaluated both across the whole cohort and, specifically, as a second-step confirmatory test in patients classified as complete responders on contrast-enhanced MRI (CE-MRI).

### 2.2. Imaging Protocol and Measurements

All MRI examinations were performed on the same 3.0 Tesla scanner using a standardised breast protocol. On pre-treatment imaging, T2-weighted signal characteristics were classified into five categories (hypointense, hyperintense, isointense, iso-hyperintense, and iso-hypointense). Tumour morphology was systematically recorded: shape (irregular or non-irregular), margin characteristics (spiculated, irregular, or other), and internal enhancement pattern (heterogeneous, homogeneous, or rim-type). The kinetic curve was classified according to late-phase signal behaviour as Type 1 (progressive increase), Type 2 (plateau), or Type 3 (washout). Cases in which signal intensity increased by ≥100% within the first two minutes after injection were designated as rapid enhancers; the remainder were classified as slow. Multifocality, multicentricity, peritumoural oedema, skin thickening, nipple involvement, chest wall involvement, and the presence of non-mass enhancement (NME) were also recorded as part of the imaging assessment.

Examinations were reviewed independently by two breast radiologists with 8 and 12 years of breast-imaging experience who were blinded to the pathological outcome, and disagreements were resolved by consensus. On diffusion-weighted imaging (DWI; b = 0 and 800 s/mm^2^), regions of interest were drawn manually within the tumour at the highest b-value, targeting the area of most restricted diffusion on a single representative slice while avoiding cystic, necrotic, and haemorrhagic areas. Pre-treatment minimum and maximum apparent diffusion coefficient values [Pre-ADC (min) and Pre-ADC (max), ×10^−6^ mm^2^/s] were measured in this way [8,9]. Inter-observer reproducibility of ADC measurements was assessed in a random subset of 50 cases, yielding an intraclass correlation coefficient of 0.91, indicating excellent agreement.

After completion of NAC, the same MRI protocol was repeated; post-treatment ADC (Post-ADC) and residual tumour long-axis diameter in millimetres were recorded. The median interval between post-NAC MRI and surgery was 21 days, with a range of 7–42 days. Two separate enhancement readings were taken from each post-treatment study, and both were made by the same two blinded radiologists in consensus. The first was a simple binary record of whether any focal contrast enhancement was visible within the tumour bed, documented as present or absent; this reading was deliberately inclusive and registered even faint, non-suspicious enhancement. MRI response was categorised as complete response, partial response, stable disease, or indeterminate; complete response was defined as the complete absence of abnormal contrast enhancement in the tumour bed, where abnormal enhancement was specified in advance as a residual enhancing mass or non-mass lesion judged suspicious for tumour at the tumour-bed site. Minimal benign background parenchymal enhancement was not counted as abnormal and did not, by itself, exclude a complete-response reading. Any residual enhancing mass or non-mass lesion meeting this threshold was classified as partial response or stable disease, and examinations in which enhancement could not be reliably characterised were classified as indeterminate. For binary comparisons, the latter three categories were combined into a single “residual disease” group. Because the binary present/absent descriptor and the response categorisation followed different rules, a tumour bed could be marked as showing enhancement on the binary descriptor and still be read as a complete responder, as long as that enhancement stayed faint and below the threshold set for abnormal residual disease. The percentage change in ADC (ΔADC) was calculated as: ΔADC (%) = [(Post-ADC − Pre-ADC (min))/Pre-ADC (min)] × 100 [9,10].

### 2.3. Surgical Management and Pathological Assessment

The NAC regimen was determined by the multidisciplinary oncology board, and all patients who completed chemotherapy proceeded to surgical resection. Patients received standard anthracycline- and taxane-based chemotherapy regimens, and HER2-positive patients received anti-HER2 therapy; dual HER2 blockade and immunotherapy were not used in this cohort. The choice between breast-conserving surgery and mastectomy was made by the board, incorporating tumour response and patient preference. All resection and axillary specimens were examined by an experienced breast pathologist following a standardised protocol. Pathologic complete response (pCR) was defined as the complete absence of residual invasive and in situ tumour in the primary tumour bed and sampled lymph nodes; residual ductal carcinoma in situ was not considered compatible with pCR (ypT0 ypN0 definition applied) [11].

### 2.4. Statistical Analysis

The distribution of continuous variables was assessed with the Shapiro–Wilk test; all continuous variables departed from normality (*p* < 0.001) and are therefore reported as a median with an interquartile range (IQR). Between-group comparisons of pCR and non-pCR cases used the Mann–Whitney U test. Categorical variables are presented as counts and percentages. Chi-square testing was applied when all expected cell counts were ≥5; Fisher’s exact test was used for 2 × 2 tables with expected counts below this threshold, including cells with zero observations, and the Freeman–Halton exact test was applied to larger contingency tables. Indeterminate examinations were grouped with residual disease for the binary analysis, and no study variable had missing data.

Throughout the analysis the target (positive) condition was pathological complete response, and an MRI complete-response reading was treated as the positive test result; sensitivity, specificity, and the predictive values are accordingly expressed with respect to pCR rather than to the detection of residual disease. The agreement between post-treatment MRI assessment and pathological outcome was quantified by sensitivity, specificity, positive predictive value (PPV), negative predictive value (NPV), and overall accuracy. Confidence intervals were derived by the Wilson score method, and categorical concordance between MRI and pathology was expressed as Cohen’s kappa coefficient. The predictive value of ADC parameters for pCR was evaluated by receiver operating characteristic (ROC) curve analysis and the area under the curve (AUC), with 95% confidence intervals estimated by 2000-iteration bootstrap resampling and optimal thresholds identified by the Youden index. The optimism of these data-derived thresholds was examined within the same bootstrap framework by computing the optimism-corrected AUC, and the stability of each cut-off was summarised as its median and interquartile range across resamples. The incremental value of post-treatment ADC beyond CE-MRI was assessed both by direct comparison of diagnostic performance and by applying the Post-ADC threshold as a sequential confirmatory step within CE-MRI complete responders. Independent predictors of pCR were evaluated by logistic regression; because only 37 patients achieved pCR, the multivariable analysis was kept deliberately parsimonious and estimated by Firth penalised regression. As ΔADC is mathematically derived from Post-ADC, the two parameters were entered into separate models, and ER and PR were not modelled together with HER2 owing to receptor-status collinearity. Because the biological heterogeneity of HER2-positive and HER2-negative subgroups may confound pre-treatment ADC performance, pre-ADC parameters were also examined by HER2-stratified ROC analysis, with pCR and non-pCR cases compared separately within each subgroup. Post-ADC and ΔADC thresholds derived from the full-cohort analysis were applied as fixed reference values in subgroup analyses. A two-tailed *p* < 0.05 was considered statistically significant throughout.

### 2.5. Ethics

The study was conducted in accordance with the Declaration of Helsinki and approved by the Institutional Ethics Committee of Adıyaman University (Protocol No: 2024/7-11; date of approval: 17 September 2024). Given the retrospective design, the requirement for individual written informed consent was waived by the ethics committee; all patient data were fully anonymised prior to analysis.

## 3. Results

### 3.1. Patient Characteristics

A total of 188 patients who received neoadjuvant chemotherapy (NAC) for breast cancer were included. Median age was 48 years, median tumour long-axis diameter 31 mm, and median Ki-67 index 18%. Oestrogen receptor (ER) positivity was present in 68.1% of patients and progesterone receptor (PR) positivity in 53.7%; HER2 positivity was recorded in 35.6%. Most tumours were irregular in shape (94.7%) with heterogeneous internal enhancement (78.2%). Pathologic complete response (pCR) was achieved in 37 patients (19.7%) (Table 1).

### 3.2. Pre-Treatment Clinical and MRI Characteristics

Several baseline features separated the pCR and non-pCR groups clearly. Median age was lower in the pCR group (45 vs. 52 years; *p* < 0.001), as was median Ki-67 (10% vs. 20%; *p* = 0.006). HER2 positivity was markedly more prevalent among pCR patients (64.9% vs. 28.5%; *p* < 0.001), while ER and PR positivity were substantially lower in this group (both *p* ≤ 0.002). Peritumoural oedema on pre-treatment MRI was far more frequent in the pCR group (70.3% vs. 34.4%; *p* < 0.001), and T2-weighted signal characteristics, tumour margin, and internal enhancement pattern also differed significantly between groups. Pre-treatment ADC parameters—minimum and maximum—carried no statistically significant predictive value in the full-cohort analysis; we attribute this to a dilution effect arising from the mixed analysis of biologically distinct HER2-positive and HER2-negative subgroups (Table 2).

### 3.3. Post-Treatment MRI Response and Diagnostic Performance

**The target condition in all of these analyses was pathological complete response, and post-treatment MRI response was scored on a single criterion: whether abnormal contrast enhancement persisted in the tumour bed.** MRI complete response was identified in 52 patients (27.7%). **Every one of the 37 women who reached pCR fell within this group, so no false-negative results arose,** and 15 patients were classified as false-positive. Sensitivity **for pCR** was 100.0% and specificity 90.1%; PPV was 71.2% and NPV 100.0%. Overall accuracy reached 92.0%, with a Cohen’s kappa of 0.781 (Table 3). For the binary comparison, the five indeterminate examinations were grouped with residual disease; each of them corresponded to residual disease at pathology, so this grouping did not alter the diagnostic estimates.

### 3.4. Post-Treatment Quantitative MRI Parameters

Median residual tumour size was 0 mm in the pCR group versus 14 mm in the non-pCR group (*p* < 0.001). Post-treatment ADC (Post-ADC) values were markedly higher in pCR patients (median 1712 vs. 1115 × 10^−6^ mm^2^/s; *p* < 0.001). The separation in ΔADC was equally striking: median 122.0% in the pCR group versus 41.4% in the non-pCR group (*p* < 0.001). Any focal tumour-bed enhancement on post-treatment imaging was nearly universal in the non-pCR group (88.1%) and rare in those who achieved pCR (16.2%; *p* < 0.001) (Table 4). This last value simply records whether any focal enhancement was visible at the tumour-bed site and is a separate reading from the criterion used to assign MRI response. In the six pCR cases concerned, that enhancement was faint and non-suspicious, never reached the threshold we had set for residual disease, and so did not pull them out of the complete-response category—which is why all 37 pCR cases remain complete responders in Table 3 even though six show enhancement in Table 4.

### 3.5. Predictive Value of ADC Parameters

**Figure 2:**
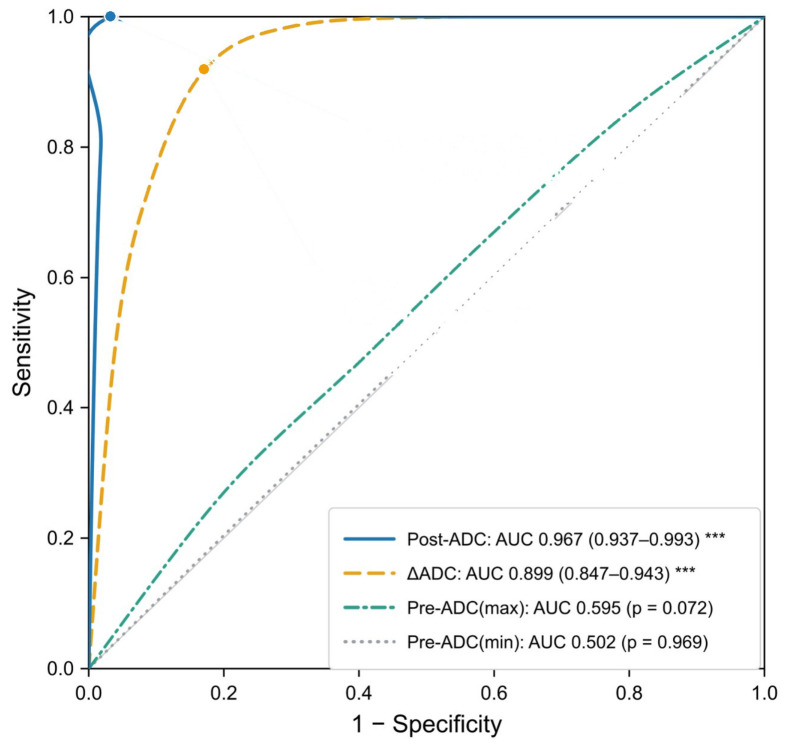
Receiver operating characteristic curves for post-treatment ADC (Post-ADC), percentage change in ADC (ΔADC), and pre-treatment ADC parameters [Pre-ADC (max) and Pre-ADC (min)] for predicting pathological complete response in the full cohort (*n* = 188). The area under the curve with a 95% confidence interval is shown for each parameter. Filled circles mark the Youden-derived operating points for the significant parameters (Post-ADC at 1513 × 10^−6^ mm^2^/s, sensitivity 100.0%, specificity 96.7%; ΔADC at 76.3%, sensitivity 91.9%, specificity 82.8%). The diagonal line denotes no discrimination. *** *p* < 0.001. ADC, apparent diffusion coefficient; AUC, area under the curve; Post-ADC, post-treatment ADC; ROC, receiver operating characteristic; ΔADC, percentage change in ADC.

Post-ADC yielded the highest discriminatory performance for pCR prediction (AUC = 0.967; 95% CI: 0.937–0.993). At a threshold of 1513 × 10^−6^ mm^2^/s, sensitivity was 100.0% and specificity 96.7%. ΔADC also performed well (AUC = 0.899; 95% CI: 0.847–0.943); at a cut-off of 76.3%, sensitivity was 91.9% and specificity 82.8%. Pre-ADC parameters (both minimum and maximum) failed to reach significance in the full-cohort analysis—a finding that reflects the heterogeneity inherent in analysing HER2-positive and HER2-negative patients together. The ROC curves for all four diffusion parameters in the full cohort are shown in Figure 2, with the corresponding values summarised in Table 5.

Because both thresholds were derived within the same cohort, we examined their internal stability by bootstrap resampling. The optimism was small: the Post-ADC AUC fell only from 0.967 to an optimism-corrected 0.958, and the ΔADC AUC from 0.899 to 0.884. The Youden-derived cut-offs were similarly stable across resamples, with a median of 1510 × 10^−6^ mm^2^/s [IQR 1492–1536] for Post-ADC and 75.8% [IQR 70.4–81.6] for ΔADC, both close to the original values (Table 6).

### 3.6. Incremental Value Beyond CE-MRI

Compared with CE-MRI alone, applying the Post-ADC threshold across the cohort increased specificity from 90.1% to 96.7% and positive predictive value from 71.2% to 88.1%, while sensitivity and negative predictive value remained at 100.0% (Table 7). To test Post-ADC specifically as a confirmatory step, we applied the threshold only within the 52 patients classified as complete responders on CE-MRI. In this subgroup it confirmed all 37 true complete responders and reclassified 10 of the 15 false-positive cases as residual disease, raising the positive predictive value from 71.2% to 88.1% without missing any true responders (Table 7). Because this confirmatory step was evaluated only within the 52 CE-MRI complete responders, its specificity (66.7%) is calculated against the 15 CE-MRI false-positive cases alone (specificity denominator = 15) and is therefore not directly comparable with the full-cohort specificity values reported for the other strategies in Table 7.

### 3.7. Independent Predictors of pCR

On univariable logistic regression, Post-ADC, ΔADC, HER2 positivity, peritumoural oedema, and any focal tumour-bed enhancement were each associated with pCR. In the reduced multivariable models, Post-ADC remained an independent predictor (adjusted OR 2.21 per 100 × 10^−6^ mm^2^/s; 95% CI 1.54–3.18; *p* < 0.001), and ΔADC retained independent value in the parallel model (adjusted OR 1.34 per 10%; 95% CI 1.13–1.60; *p* = 0.001). HER2 positivity remained positively associated and any focal tumour-bed enhancement inversely associated with pCR (Table 8).

### 3.8. Subgroup Analysis by HER2 Status

**Figure 3:**
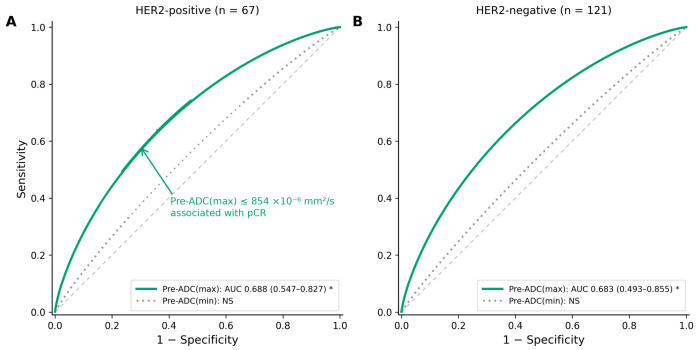
ROC curves of pre-treatment ADC parameters for predicting pathological complete response by HER2 status (Panel (**A**), HER2-positive, *n* = 67; Panel (**B**), HER2-negative, *n* = 121). AUC with 95% CI is shown for Pre-ADC (max); Pre-ADC (min) was not significant in either subgroup. In HER2-positive tumours, Pre-ADC (max) ≤ 854 × 10^−6^ mm^2^/s was associated with pCR (**A**). The diagonal line denotes no discrimination (**B**). * *p* < 0.05. ADC, apparent diffusion coefficient; AUC, area under the curve; HER2, human epidermal growth factor receptor 2; NS, not significant; pCR, pathological complete response.

The pCR rate differed substantially by HER2 status, reaching 35.8% in HER2-positive and 10.7% in HER2-negative tumours (Table 9). In the subgroup analysis, Pre-ADC (max) reached statistical significance in both HER2-positive (AUC = 0.688; 95% CI: 0.547–0.827; *p* = 0.011) and HER2-negative (AUC = 0.683; 95% CI: 0.493–0.855; *p* = 0.032) patients, whereas Pre-ADC (min) did not reach the significance threshold in either group. An inverse relationship was observed in HER2-positive patients: Pre-ADC (max) values at or below 854 × 10^−6^ mm^2^/s were associated with pCR (Figure 3).

## 4. Discussion

The question of whether post-NAC imaging can reliably confirm pCR has gained clinical urgency as de-escalation strategies—omitting axillary dissection, reducing adjuvant therapy—become increasingly tied to response assessment. We raise this only as the clinical context that motivated the study; our single-centre findings are not in themselves grounds for any such de-escalation, which would first require prospective, externally validated evidence. Our findings reinforce CE-MRI’s strength as a rule-out tool while exposing the limits that make it insufficient as a standalone arbiter of pCR. The 15 false-positive cases are not a marginal finding; they mean that roughly one in three patients whom MRI labelled as complete responders still harboured residual disease at pathology. That gap is where Post-ADC and ΔADC matter most, and it is the gap we set out to close: applied to these same complete responders—the 52 patients whose tumour beds showed no abnormal enhancement on CE-MRI—a post-treatment ADC threshold reclassified 10 of the 15 false positives as residual disease while keeping every true responder correctly identified (Table 7).

Post-treatment CE-MRI achieved 100% sensitivity and 100% NPV, placing it well above the pooled sensitivity of 0.83 reported in the broader literature—no true pCR case was missed [12]. Our specificity (90.1%) and accuracy (92.0%) were close to those reported by Zhang et al. in a series of 177 patients (97.44% and 93.79%, respectively), though our PPV (71.2%) fell somewhat short of their 77.78% [13]. The Cohen’s kappa of 0.781 exceeded both the 0.60 recorded in the non-immunotherapy triple-negative breast cancer (TNBC) subgroup and the overall cohort value of 0.57 in Dhungana et al., suggesting that MRI–pathology concordance may be stronger in standard chemotherapy-treated populations unaffected by immunotherapy-related inflammatory changes [14]. Yet the PPV of 71.2% is hard to ignore: nearly three in ten MRI-designated complete responders did not achieve pCR. Wu et al. reported that a biologically based model built on quantitative MRI raised specificity from 79%, the value obtained with tumour volume alone, to 95% [15], and our data point the same way: pairing CE-MRI with a diffusion measurement recovers much of the specificity that imaging morphology by itself leaves behind.

The biological basis for post-treatment ADC elevation in pCR is well established—tumour cell death increases extracellular free water, driving diffusivity upward. Our results confirm this clearly. Median Post-ADC in pCR patients reached 1712 × 10^−6^ mm^2^/s versus 1115 × 10^−6^ mm^2^/s in non-pCR patients, and Post-ADC achieved an AUC of 0.967, the highest in this study. The post-treatment ADC values we measured fall in the range reported by Partridge et al., whose whole-tumour ADC reached a mean of about 1.62 × 10^−3^ mm^2^/s at the post-treatment time point, even though ADC alone discriminated only modestly in that multicentre trial [16]. That modest stand-alone performance has motivated more elaborate diffusion models, such as the restriction spectrum imaging approach of Andreassen et al., intended to sharpen a response signal that a single ADC threshold can miss [17]. We attribute the gap between those reports and ours to ROI methodology, b-value selection, and the measurement noise introduced by multicentre technical heterogeneity, all of which tend to blunt ADC’s discriminatory signal. An AUC of 0.967 is unusually high for this task, and we treat it with caution: the threshold was both derived and tested in the same cohort, so some optimism is unavoidable. Bootstrap optimism correction lowered it only slightly, to 0.958, and the cut-off was stable across resamples (Table 6); after adjustment, Post-ADC also remained an independent predictor of pCR (Table 8). These checks are reassuring, but they are internal—not a substitute for validation in an independent cohort.

For ΔADC, our optimal threshold of 76.3% substantially exceeds the mid-treatment 50% increase reported in the multicentre series by Partridge et al. [16]. The difference is consistent with two factors: the measurement consistency that comes with a single-centre protocol, and the more complete tissue response expected at the post-treatment time point compared with interim imaging. Suo et al. found that diffusion change alone predicted pCR with an AUC in the region of 0.75–0.83, rising to 0.905 once oestrogen-receptor and HER2 status were added [18]. Our data fit the same picture: any focal tumour-bed enhancement was present in 88.1% of non-pCR cases but only 16.2% of pCR cases, adding information beyond Post-ADC alone. Liang et al. similarly reported that appending DCE-MRI parameters to ADC increased specificity from 56.84% to 95.79%, reinforcing the case for multiparametric rather than single-parameter assessment [19].

HER2 positivity was the strongest baseline predictor of pCR in our cohort (64.9% vs. 28.5%; *p* < 0.001). This rate aligns closely with the 61.5% reported by Jung et al. in a purely HER2-positive series and the 62.2% recorded by Falcón González et al. across 310 HER2-positive patients in a real-world setting [20,21]. The association between hormone receptor negativity and pCR followed the expected direction; in Fang et al.’s series of 279 patients, the ER/PR-negative HER2-positive subtype reached a pCR rate of 45.83%, whereas the ER/PR-positive HER2-negative group reached only 7.8% [22].

The Ki-67 finding deserves pause. Median Ki-67 was lower in pCR patients (10% vs. 20%), which may seem counterintuitive. Jung et al. found no significant relationship between Ki-67 and pCR in their HER2-positive series (*p* = 0.743), and the most plausible interpretation is that HER2-directed therapy drives response independently of the proliferation index, while high-Ki-67 luminal B tumours in the non-pCR group inflate the group median [20]. Falcón González et al. identified Ki-67 >20% as an independent predictor in a mixed HER2-positive series but explicitly cautioned that this relationship cannot be disentangled from hormone receptor status [21]. In the HR-positive/HER2-negative subset, Ki-67 lost independent predictive value on multivariate analysis—consistent with Topal and Başak’s findings—with low ER percentage emerging as the more decisive variable [23]. The association between younger age and pCR (median 45 vs. 52 years; *p* < 0.001) diverges from Fang et al.’s data, and we suspect this reflects the relatively higher proportion of HER2-positive and triple-negative tumours among younger patients in our cohort rather than age as an independent biological determinant [22].

Peritumoural oedema was markedly more prevalent in pCR patients (70.3% vs. 34.4%; *p* < 0.001), a finding consistent with its proposed biological basis in immune activation and increased vascular permeability. Kwon et al. identified post-NAC peritumoural oedema on MRI as the strongest independent prognostic factor for both distant metastasis-free survival and overall survival in luminal breast cancer, with a prognostic separation that exceeded that of pCR itself [24]. Tumour margin, internal enhancement pattern, and T2-weighted signal characteristics also differed significantly between groups. The complete absence of nipple involvement in pCR patients (0% vs. 14.6%; *p* = 0.009) points to a consistent negative association that may reflect more locally advanced disease in that subgroup. Heacock et al., working exclusively with HER2-positive patients, found that neither internal enhancement type (*p* = 0.136) nor lesion shape (*p* = 0.391) predicted pCR, reinforcing the view that morphological features carry subtype-dependent weight [25]. Multifocality, multicentricity, skin thickening, chest wall involvement, NME, kinetic curve type, and enhancement rate showed no association with pCR in our analysis—a finding that narrows the pre-treatment MRI features with genuine discriminatory value.

Pre-ADC (min) performed near chance (AUC = 0.502; *p* = 0.969), and Pre-ADC (max) failed to reach significance (AUC = 0.595; *p* = 0.072) in the full-cohort analysis. Musall et al. similarly found pre-treatment tumour ADC values non-predictive of pCR in a TNBC series, and we interpret both results as a dilution effect: combining HER2-positive and HER2-negative tumours—which carry biologically distinct ADC profiles—in a single analysis obscures any subtype-specific signal [26]. Lin et al. confirmed the ceiling of single-modality morphological assessment by showing that adding radiomic parameters to clinicopathological features raised pCR prediction AUC from 0.689 to 0.852 [27].

A finding that runs against the usual direction deserves comment. In the HER2-positive subgroup, it was a lower, not a higher, pre-treatment ADC that tracked with pCR, with Pre-ADC (max) values at or below 854 × 10^−6^ mm^2^/s associated with response (Table 9, Figure 3A). A low ADC reflects dense cellularity and restricted water diffusion, and the most plausible interpretation is that the densely cellular, highly proliferative HER2-positive tumours—those expected to respond best to anthracycline–taxane chemotherapy combined with HER2-directed therapy—are the same tumours that go on to achieve pCR, so that a low baseline ADC marks chemosensitive biology rather than resistance. This reading remains speculative. The subgroup is small (*n* = 67, with 24 pCR events), the confidence interval around the AUC is wide (0.688, 95% CI 0.547–0.827), and the threshold was both identified and tested in the same cohort. We therefore report the inverse Pre-ADC (max) association as a hypothesis-generating observation that needs confirmation in independent, prospectively assembled HER2-positive cohorts before it can be treated as a reliable predictor.

The subtype dependence of pre-treatment ADC raises a fair question about our post-treatment threshold: can one Post-ADC cut-off reasonably be applied across molecular subtypes? Our data can raise this question but cannot answer it. The predictive signal in pre-treatment ADC was subtype-specific—Pre-ADC (max) reached significance only after HER2 stratification (Table 9)—whereas post-treatment ADC reflects something more uniform, namely the rise in free water that follows tumour cell death, a downstream marker of response rather than a fingerprint of the original tumour biology. In the cohort as a whole, the single Post-ADC threshold identified the true responders without an obvious subtype-related miss; we deliberately stop short of reading this as a subtype-level result. With only 37 pCR events divided between HER2-positive (*n* = 24) and HER2-negative (*n* = 13) tumours, the subgroups are far too small to support a firm conclusion, and this observation should be treated as hypothesis-generating rather than as evidence that one threshold performs equally well in every subtype. Zhang et al. showed that MRI accuracy varies by subtype, with the highest predictive value in TNBC and Luminal B tumours [13], and Partridge et al. documented subtype-dependent differences in the time course of ADC response [17]. A single threshold may thus prove convenient for confirming response, but whether it is truly adequate within each subtype—or instead needs subtype-specific calibration—is a question our data cannot settle, since we neither derived nor validated separate thresholds within the subgroups.

The retrospective single-centre design is the principal constraint on this study’s generalisability. Referral patterns and institutional selection criteria may not reflect the broader NAC-treated population, and the ADC measurement protocol—specific b-value selection and manual ROI placement—limits direct numerical comparability with studies using different acquisition parameters. A second limitation is statistical. Both the Post-ADC and ΔADC thresholds were derived and tested in the same patients, which inflates their apparent performance; we addressed this with bootstrap optimism correction, and the corrected values stayed close to the originals (Table 6), but no external cohort has yet confirmed them, and the AUC of 0.967 should be read in that light rather than as a transportable figure. Third, only 37 patients achieved pCR. This small number of events limits how many covariates a regression can responsibly carry, so we kept the multivariable models parsimonious, applied Firth penalisation, and entered collinear predictors separately (Table 8); even so, the adjusted estimates are better regarded as exploratory than as definitive. Fourth, a single Post-ADC threshold was applied to every subtype, and subtype-specific cut-offs—which a larger cohort would allow—were not derived. What the single-centre design does provide is technical consistency, which likely contributes to the tight ADC thresholds we identified. The cohort’s restriction to standard chemotherapy regimens, with immunotherapy and dual HER2 blockade excluded, removes a source of imaging interpretation noise that has complicated other series. Whether the HER2-stratified ADC behaviour and the sequential confirmation rule we describe hold up in larger, subtype-balanced cohorts assembled prospectively across multiple centres remains to be established; until then, our results are best read as a well-characterised single-centre signal rather than a validated decision rule.

## 5. Conclusions

Post-NAC CE-MRI is a dependable rule-out test for pathological complete response—in our cohort it missed no true pCR, reaching 100% sensitivity and 100% negative predictive value—but its false-positive rate means it cannot, on its own, confirm that a complete response has been achieved. In our cohort, post-treatment ADC and ΔADC helped distinguish the true complete responders from these false positives—the point at which morphological assessment alone tends to fall short. We present this as a diagnostic observation rather than a change in practice. The thresholds were derived and tested within a single retrospective, single-centre cohort and checked only by internal resampling; they have not been confirmed in any external population, and the values we report—including the Post-ADC AUC of 0.967—should be regarded as cohort-specific rather than transportable. They are therefore not yet a basis for surgical or adjuvant decisions on their own. Establishing whether quantitative diffusion can be used in this way will require prospective, multicentre validation, ideally with subtype-specific thresholds derived and tested within each subgroup.

## Figures and Tables

**Table 1:** Baseline clinical and pre-treatment MRI characteristics of the study population (*n* = 188).

Parameter	*n* (%) or Median [IQR]
**Age, years**	48 [42–56]
**Tumour long-axis diameter, mm**	31 [25–39]
**Ki-67, %**	18 [10–40]
**Pre-ADC (max), ×10^−6^ mm^2^/s**	972 [854–1001]
**Pre-ADC (min), ×10^−6^ mm^2^/s**	818 [762–930]
**ER positivity**	128 (68.1)
**PR positivity**	101 (53.7)
**HER2 positivity**	67 (35.6)
**T2-weighted signal—hypointense**	84 (44.7)
**T2-weighted signal—hyperintense**	35 (18.6)
**T2-weighted signal—isointense**	34 (18.1)
**T2-weighted signal—iso-hyperintense**	18 (9.6)
**T2-weighted signal—iso-hypointense**	17 (9.0)
**Tumour shape—irregular**	178 (94.7)
**Tumour margin—spiculated**	107 (56.9)
**Tumour margin—irregular**	59 (31.4)
**Tumour margin—other**	22 (11.7)
**Internal enhancement —heterogeneous**	147 (78.2)
**Internal enhancement—homogeneous**	36 (19.1)
**Internal enhancement—rim-type**	5 (2.7)
**Kinetic curve—type 1**	68 (36.2)
**Kinetic curve—type 2**	105 (55.9)
**Kinetic curve—type 3**	15 (8.0)
**Enhancement rate—rapid**	120 (63.8)
**Multifocality**	30 (16.0)
**Multicentricity**	39 (20.7)
**Peritumoural oedema**	78 (41.5)
**Skin thickening**	42 (22.3)
**Nipple involvement**	22 (11.7)
**Chest wall involvement**	5 (2.7)
**Non-mass enhancement**	14 (7.4)
**MRI response—partial**	115 (61.2)
**MRI response—complete**	52 (27.7)
**MRI response—stable disease**	16 (8.5)
**MRI response—indeterminate**	5 (2.7)
**Pathological complete response**	37 (19.7)

**Abbreviations and notes:** Data are median [IQR] or *n* (%); all continuous variables were non-normal on the Shapiro–Wilk test (*p* < 0.001). ADC, apparent diffusion coefficient; ER, oestrogen receptor; HER2, human epidermal growth factor receptor 2; IQR, interquartile range; MRI, magnetic resonance imaging; PR, progesterone receptor.

**Table 2:** Pre-treatment clinical and MRI characteristics according to pathological response group (*n* = 188).

Parameter	pCR (*n* = 37)	Non-pCR (*n* = 151)	*p*
**Age, years ^a^**	45 [34–47]	52 [44–58]	<0.001
**Tumour long-axis diameter, mm ^a^**	38 [24–47]	30 [25–38]	0.066
**Ki-67, % ^a^**	10 [10–25]	20 [10–45]	0.006
**Pre-ADC (max), ×10^−6^ mm^2^/s ^a^**	893 [801–970]	985 [867–1001]	0.072
**Pre-ADC (min), ×10^−6^ mm^2^/s ^a^**	783 [771–887]	818 [762–931]	0.969
**ER positivity ^b^**	11 (29.7)	117 (77.5)	<0.001
**PR positivity ^b^**	11 (29.7)	90 (59.6)	0.002
**HER2 positivity ^b^**	24 (64.9)	43 (28.5)	<0.001
**T2-weighted signal ^c^**	—	—	0.005
**hypointense**	18 (48.6)	66 (43.7)	
**isointense**	13 (35.1)	21 (13.9)	
**hyperintense**	3 (8.1)	32 (21.2)	
**iso-hyperintense**	0 (0.0)	18 (11.9)	
**iso-hypointense**	3 (8.1)	14 (9.3)	
**Multifocality ^b^**	5 (13.5)	25 (16.6)	0.840
**Multicentricity ^b^**	7 (18.9)	32 (21.2)	0.937
**Peritumoural oedema ^b^**	26 (70.3)	52 (34.4)	<0.001
**Skin thickening ^b^**	12 (32.4)	30 (19.9)	0.154
**Nipple involvement ^d^**	0 (0.0)	22 (14.6)	0.009
**Chest wall involvement ^d^**	0 (0.0)	5 (3.3)	0.585
**Non-mass enhancement ^d^**	5 (13.5)	9 (6.0)	0.147
**Tumour shape—irregular ^d^**	32 (86.5)	146 (96.7)	0.038
**Tumour margin ^c^**	—	—	0.003
**spiculated**	13 (35.1)	94 (62.3)	
**irregular**	15 (40.5)	44 (29.1)	
**other**	9 (24.3)	13 (8.6)	
**Internal enhancement ^c^**	—	—	0.002
**heterogeneous**	37 (100.0)	110 (72.8)	
**homogeneous**	0 (0.0)	36 (23.8)	
**rim-type**	0 (0.0)	5 (3.3)	
**Kinetic curve ^c^**	—	—	0.230
**type 1**	10 (27.0)	58 (38.4)	
**type 2**	22 (59.5)	83 (55.0)	
**type 3**	5 (13.5)	10 (6.6)	
**Enhancement rate—rapid ^b^**	27 (73.0)	93 (61.6)	0.271

**Abbreviations and notes:** Data are *n* (%) or median [IQR]. ^a^ Mann–Whitney U test; ^b^ chi-square test; ^c^ Freeman–Halton exact test; ^d^ Fisher’s exact test. ADC, apparent diffusion coefficient; ER, oestrogen receptor; HER2, human epidermal growth factor receptor 2; IQR, interquartile range; pCR, pathological complete response; PR, progesterone receptor.

**Table 3:** Diagnostic performance of post-treatment MRI response assessment against surgical pathology.

Post-Treatment MRI	Pathology: pCR (*n* = 37)	Pathology: Non-pCR (*n* = 151)	Total
**Complete response**	37	15	52
**Residual disease ^a^**	0	136	136
**Total**	37	151	188
**Diagnostic measure**	**Value, % (95% CI)**		
**Sensitivity**	100.0 (90.6–100.0)		
**Specificity**	90.1 (84.3–93.9)		
**Positive predictive value**	71.2 (57.7–81.7)		
**Negative predictive value**	100.0 (97.3–100.0)		
**Accuracy**	92.0 (87.3–95.1)		
**Cohen’s κ**	0.781		

Abbreviations and notes: Counts are referenced against surgical pathology. ^a^ Partial response, stable disease, and indeterminate categories were grouped as residual disease; each indeterminate examination corresponded to residual disease at pathology. MRI complete response denotes the complete absence of abnormal contrast enhancement in the tumour bed, where abnormal enhancement is defined a priori as a residual enhancing mass or non-mass enhancement at the tumour-bed site; minimal benign background parenchymal enhancement was not classified as abnormal and did not preclude a complete-response designation. Confidence intervals were derived by the Wilson score method. CI, confidence interval; MRI, magnetic resonance imaging; pCR, pathological complete response.

**Table 4:** Post-treatment quantitative MRI parameters according to pathological response group.

Parameter	pCR (*n* = 37)	Non-pCR (*n* = 151)	*p*
**Residual tumour size, mm ^a^**	0 [0–0]	14 [10.5–21.0]	<0.001
**Post-ADC, ×10^−6^ mm^2^/s ^a^**	1712 [1703–1770]	1115 [1081–1216]	<0.001
**ΔADC, % ^a^**	122.0 [85.7–127.7]	41.4 [19.9–63.7]	<0.001
**Any focal tumour-bed enhancement ^b^**	6 (16.2)	133 (88.1)	<0.001

Abbreviations and notes: Data are median [IQR] or *n* (%). ^a^ Mann–Whitney U test; ^b^ Fisher’s exact test. ΔADC was calculated as [(Post-ADC − Pre-ADC (min))/Pre-ADC (min)] × 100. Any focal tumour-bed enhancement is a binary present/absent descriptor recorded at the tumour-bed site on post-treatment imaging and is distinct from the abnormal-enhancement criterion used to define MRI complete response; faint or non-suspicious enhancement recorded as present under this descriptor did not meet the threshold for residual disease and was therefore compatible with an MRI complete-response designation. ADC, apparent diffusion coefficient; IQR, interquartile range; pCR, pathological complete response; Post-ADC, post-treatment apparent diffusion coefficient; ΔADC, percentage change in apparent diffusion coefficient.

**Table 5:** Diagnostic performance of diffusion parameters for predicting pathological complete response (*n* = 188).

Parameter	AUC	95% CI	Cut-Off (×10^−6^ mm^2^/s or %)	Sensitivity, %	Specificity, %	PPV, %	NPV, %	*p*
**Post-ADC**	0.967	0.937–0.993	1513	100.0	96.7	88.1	100.0	<0.001
**ΔADC**	0.899	0.847–0.943	76.3	91.9	82.8	56.7	97.7	<0.001
**Pre-ADC (max)**	0.595	—	—	—	—	—	—	0.072
**Pre-ADC (min)**	0.502	—	—	—	—	—	—	0.969

Abbreviations and notes: AUC and 95% CI were obtained by ROC analysis with bootstrap resampling (2000 iterations); cut-offs were set by the Youden index. PPV and NPV were calculated at the observed pCR prevalence of 19.7%. Cut-off, sensitivity, specificity, PPV, and NPV are given only for significant parameters. ADC, apparent diffusion coefficient; AUC, area under the ROC curve; CI, confidence interval; NPV, negative predictive value; pCR, pathological complete response; PPV, positive predictive value; ΔADC, percentage change in apparent diffusion coefficient.

**Table 6:** Internal validation and threshold stability of diffusion parameters for predicting pathological complete response.

Parameter	Apparent AUC (95% CI)	Optimism-Corrected AUC	Mean Optimism	Original Cut-Off	Bootstrap Cut-Off, Median [IQR]
**Post-ADC**	0.967 (0.937–0.993)	0.958	0.009	1513 × 10^−6^ mm^2^/s	1510 [1492–1536] × 10^−6^ mm^2^/s
**ΔADC**	0.899 (0.847–0.943)	0.884	0.015	76.3%	75.8 [70.4–81.6]%

Abbreviations and notes: Optimism-corrected AUC and cut-off stability (median [IQR]) were obtained by bootstrap resampling (2000 iterations). AUC, area under the ROC curve; CI, confidence interval; IQR, interquartile range; Post-ADC, post-treatment apparent diffusion coefficient; ΔADC, percentage change in apparent diffusion coefficient.

**Table 7:** Comparative diagnostic performance of post-treatment imaging strategies for predicting pathological complete response.

Strategy	Analysis Set	TP	FP	FN	TN	Sensitivity, % (95% CI)	Specificity, % (95% CI)	PPV, % (95% CI)	NPV, % (95% CI)	Accuracy, % (95% CI)
**CE-MRI complete response**	Full cohort, *n* = 188	37	15	0	136	100.0 (90.6–100.0)	90.1 (84.3–93.9)	71.2 (57.7–81.7)	100.0 (97.3–100.0)	92.0 (87.3–95.1)
**Post-ADC ≥ 1513 × 10^−6^ mm^2^/s**	Full cohort, *n* = 188	37	5	0	146	100.0 (90.6–100.0)	96.7 (92.5–98.6)	88.1 (75.0–94.8)	100.0 (97.4–100.0)	97.3 (93.9–98.9)
**ΔADC ≥ 76.3%**	Full cohort, *n* = 188	34	26	3	125	91.9 (78.7–97.2)	82.8 (76.0–88.0)	56.7 (44.1–68.4)	97.7 (93.3–99.2)	84.6 (78.7–89.0)
**Post-ADC ≥ 1513 × 10^−6^ mm^2^/s within CE-MRI complete responders**	CE-MRI complete-response subgroup, *n* = 52	37	5	0	10	100.0 (90.6–100.0)	66.7 **^a^** (41.7–84.8)	88.1 (75.0–94.8)	100.0 (72.2–100.0)	90.4 (79.4–95.8)

Abbreviations and notes: Counts are referenced against surgical pathology. The first three rows report diagnostic performance in the full cohort of 188 patients (37 pathological complete responses and 151 cases with residual disease). The final row reports the sequential confirmation step among the 52 patients classified as complete responders on CE-MRI; within this subgroup, Post-ADC ≥ 1513 × 10^−6^ mm^2^/s confirmed all 37 pathological complete responses and reclassified 10 of 15 CE-MRI false-positive cases as residual disease. Confidence intervals were calculated by the Wilson score method. ^a^ Within the CE-MRI complete-response subgroup (*n* = 52), specificity is calculated against the 15 CE-MRI false-positive cases only (specificity denominator = 15; TN = 10, FP = 5), rather than against the full residual-disease denominator of 151 used in the first three rows; the specificity in this row is therefore not directly comparable with the specificity values above it. For the same reason, the PPV, NPV, and accuracy in this row are also computed within the 52-patient subgroup. ADC, apparent diffusion coefficient; CE-MRI, contrast-enhanced magnetic resonance imaging; CI, confidence interval; FN, false negative; FP, false positive; NPV, negative predictive value; Post-ADC, post-treatment apparent diffusion coefficient; PPV, positive predictive value; TN, true negative; TP, true positive; ΔADC, percentage change in apparent diffusion coefficient.

**Table 8:** Univariable and reduced penalised multivariable logistic regression models for predictors of pathological complete response (*n* = 188).

Variable	Unadjusted OR (95% CI)	*p*	Adjusted OR, Primary Post-ADC Model (95% CI)	*p*	Adjusted OR, Sensitivity ΔADC Model (95% CI)	*p*
**HER2 positivity**	4.64 (2.17–9.94)	<0.001	2.86 (1.17–7.01)	0.021	3.12 (1.31–7.44)	0.010
**ER positivity**	0.12 (0.06–0.27)	<0.001	—	—	—	—
**PR positivity**	0.29 (0.13–0.62)	0.002	—	—	—	—
**Peritumoural oedema**	4.50 (2.06–9.83)	<0.001	2.31 (0.96–5.58)	0.062	2.58 (1.09–6.12)	0.031
**Any focal tumour-bed enhancement**	0.03 (0.01–0.07)	<0.001	0.16 (0.04–0.67)	0.012	0.10 (0.03–0.39)	0.001
**Post-ADC, per 100 × 10^−6^ mm^2^/s**	2.84 (2.13–3.79)	<0.001	2.21 (1.54–3.18)	<0.001	—	—
**ΔADC, per 10%**	1.58 (1.34–1.86)	<0.001	—	—	1.34 (1.13–1.60)	0.001
**Ki-67, per 10%**	0.78 (0.66–0.92)	0.004	—	—	—	—
**Age, per year**	0.90 (0.86–0.94)	<0.001	—	—	—	—

**Notes:** Continuous-variable odds ratios are per unit increase as indicated. Adjusted models used Firth penalised logistic regression; Post-ADC and ΔADC were modelled separately. Each adjusted model included HER2 positivity, peritumoural oedema, **any focal tumour-bed enhancement**, and one diffusion parameter. CI, confidence interval; ER, oestrogen receptor; HER2, human epidermal growth factor receptor 2; OR, odds ratio; Post-ADC, post-treatment apparent diffusion coefficient; PR, progesterone receptor; ΔADC, percentage change in apparent diffusion coefficient.

**Table 9:** Pathological complete response and pre-treatment ADC performance stratified by HER2 status.

HER2 Status	*n*	pCR, *n* (%)	Pre-ADC (max) AUC	95% CI	*p*
**HER2-positive**	67	24 (35.8)	0.688	0.547–0.827	0.011
**HER2-negative**	121	13 (10.7)	0.683	0.493–0.855	0.032

**Abbreviations and notes:** The pCR rate differed between HER2 subgroups (chi-square test, *p* < 0.001). Pre-ADC (min) was not significant in either subgroup. In HER2-positive tumours, Pre-ADC (max) ≤ 854 × 10^−6^ mm^2^/s was associated with pCR. ADC, apparent diffusion coefficient; AUC, area under the ROC curve; CI, confidence interval; HER2, human epidermal growth factor receptor 2; pCR, pathological complete response.

## Data Availability

Data used in this study can be provided upon reasonable request.

## Abbreviations

The following abbreviations are used in this manuscript:

**Abbreviation**	**Full Term**
ADC	Apparent diffusion coefficient
AUC	Area under the curve
CE-MRI	Contrast-enhanced magnetic resonance imaging
CI	Confidence interval
DCE-MRI	Dynamic contrast-enhanced magnetic resonance imaging
DWI	Diffusion-weighted imaging
ER	Oestrogen receptor
FN	False negative
FP	False positive
HER2	Human epidermal growth factor receptor 2
IQR	Interquartile range
MRI	Magnetic resonance imaging
NAC	Neoadjuvant chemotherapy
NME	Non-mass enhancement
NPV	Negative predictive value
pCR	Pathologic complete response
Post-ADC	Post-treatment apparent diffusion coefficient
PPV	Positive predictive value
PR	Progesterone receptor
ROC	Receiver operating characteristic
TN	True negative
TNBC	Triple-negative breast cancer
TP	True positive
ΔADC	Percentage change in apparent diffusion coefficient
